# Research on Laser Cleaning of Graphite Lubrication Coating on the Magnesium Alloy Surface

**DOI:** 10.3390/ma18030484

**Published:** 2025-01-21

**Authors:** Zhenhai Xu, Yunhui Yue, Donghe Zhang, Shaoxi Xue, Erju Liu, Debin Shan, Jie Xu, Bin Guo

**Affiliations:** 1 CGN-HIT Advanced Nuclear and New Energy Research Institute, Harbin Institute of Technology, Harbin 150001, China; zhenhaixu@hit.edu.cn; 2 Zhengzhou Research Institute, Harbin Institute of Technology, Zhengzhou 450000, China; zhangdonghe@hit.edu.cn (D.Z.); xjhit@hit.edu.cn (J.X.); bguo@hit.edu.cn (B.G.); 3 National Key Laboratory for Precision Hot Processing of Metals, Harbin Institute of Technology, Harbin 150001, China; 22s009106@stu.hit.edu.cn (Y.Y.); xueshaoxi@hit.edu.cn (S.X.); 20b909103@stu.hit.edu.cn (E.L.)

**Keywords:** laser cleaning, graphite lubrication coating, removal mechanism, surface morphology, roughness, magnesium alloy

## Abstract

The lubricating coating must be removed from the forged or stamped workpieces. Developing environment-friendly and high-precision cleaning technology is necessary. In this study, a nanosecond pulsed laser was used to clean the graphite lubricating coating of 15 μm thickness on the surface of an MB15 magnesium alloy. The effects of various laser cleaning parameters on the cleaning quality and the cleaning mechanism were studied. When the laser fluence (*F*) increases from 1.27 to 7.64 J/cm^2^, the clearance rate increases, and the surface roughness initially decreases before increasing. When the pulse frequency (*f*) increases from 10 to 30 kHz, the single-pulse energy decreases, the clearance rate decreases, and the surface roughness increases. When the scanning speed (*v*) increases from 1000 to 5000 mm/s, the spot overlap rate decreases, the clearance rate decreases, and the surface roughness firstly decreases and then increases. The optimal cleaning parameter combinations are *F* = 3.82 J/cm^2^, *f* = 10 kHz, and *v* = 3000 mm/s. The graphite lubrication coating was almost completely removed without damaging the substrate surface, and the surface carbon content of the sample was decreased to 6.42%. The laser cleaning mechanism of the graphite lubricating coating on the magnesium alloy surface is dominated by thermal ablation. As the laser fluence increases, the physical and chemical reactions become more violent.

## 1. Introduction

During the forging or stamping process, the friction between the workpiece and the mold can lead to the difficulty of forming, the increased wear, and the premature failure of the mold. In order to enhance the product quality, minimize the wear, and prolong the mold life, utilizing high-efficacy lubricants like graphite and molybdenum disulfide is a common practice. However, after forming, they often adhere to the surface of the workpiece and need to be removed further. The traditional chemical cleaning method [1,2,3,4] utilizes chemical reactions to decrease the adhesion between dirt and the substrate. However, the prolonged use of chemical agents can corrode the substrate surface, leading to environmental pollution from the resulting waste liquid. The mechanical cleaning method [5,6,7] utilizes the friction and shear effects of abrasives on the dirt coating for removal. However, it is limited in cleaning complex-shaped parts, and the cleaning accuracy is uncontrollable, making automation challenging. The ultrasonic cleaning method [8,9] cannot effectively clean submicron particles due to its inherent limitations. Against the backdrop of vigorously promoting the transformation and upgrading of the manufacturing industry globally, traditional cleaning methods can no longer meet the high-precision and pollution-free requirements of various fields for cleaning processes. Based on this, finding an efficient method to remove the lubricant adhered to the surface of the workpiece in an environmentally friendly manner without damaging the substrate is a novel concept for the surface cleaning of forgings.

Laser cleaning, as a new type of cleaning technology, is considered the most promising green cleaning technology of the 21st century [10,11,12,13]. It has the advantages of controllability, efficacy, wide applicability, non-contact operation, and environmentally friendly green protection. In 1969, the concept of laser cleaning was initially proposed by American scientists S. M. Bedair and Harold P. Smith, Jr. They successfully removed the oxygen or sulfur contamination from the surface of nickel by exposing it to high-power laser pulses (120 MW/cm^2^). This laser-based cleaning method has served as a source of inspiration for researchers, leading to extensive and profound investigations into laser technology and its underlying mechanisms [14].

At present, laser cleaning technology has been applied in metal surface treatment (removal of oxide film [15,16,17], paint [18,19,20], rust [21,22]), semiconductor industry [23,24,25], and for cultural relic protection [26,27,28]. Zhang et al. [29] used a laser to remove the oxide film from the surface of 5754 aluminum alloy and found that the oxide coating could be entirely eliminated by the laser. The researchers explained the cleaning mechanism and plasma behavior by comparing the cleaning efficacy on aluminum alloys at various energy densities. Zhang et al. [30] used a nanosecond pulsed laser to remove the blue paint coating on an Al-Mg aluminum alloy substrate and found that the thickness of the removed coating gradually increased with low laser irradiation. The main mechanisms of blue coating detachment from the surface are ablation, peeling, and vaporization. Wang et al. [31] conducted laser cleaning experiments on the surface rust of steel using pulsed Nd: YAG and found that under appropriate laser parameters, the pulsed Nd: YAG laser can completely remove the rust coating. Moreover, the microhardness and corrosion resistance of the surface improved after laser cleaning, with the enhancement correlating with the increase in laser energy. Ye et al. [32] used Nd: YAG laser to induce optical breakdown of the airborne above the gold-coated K9 glass surface, and the created shockwave removed the SiO2 particles contaminated on the gold films. The results showed that the 1064 nm laser-induced plasma shockwave can effectively remove the SiO2 particles; the removal ratio can reach above 90%. In summary, laser cleaning can achieve high-quality cleaning efficacy for various objects without damaging the substrate by utilizing a suitable combination of laser parameters. For a specific cleaning task, the influence of laser parameters on contaminant removal can be investigated by analyzing experimental data from laser cleaning, optimizing process parameters, and understanding the characteristics of laser cleaning. Therefore, laser cleaning technology provides a new option for removing lubricating coatings from the surface of formed specimens.

So far, there are few reports on the use of laser cleaning technology for removing lubricating coatings on the surface of formed specimens. Therefore, exploring this research is of great significance. This study utilized a nanosecond pulsed laser cleaning system to conduct experiments on removing the graphite lubrication coating from the surface of MB15 magnesium alloy. The study investigated the impact of laser fluence, pulse frequency, and scanning speed on the cleaning efficacy. This study evaluated the surface characteristics of the cleaned samples using optical microscopy, scanning electron microscopy (SEM), energy dispersive spectroscopy (EDS), and laser confocal microscopy. It also explored the mechanism of laser cleaning of the graphite coating by employing a high-speed camera to observe the dynamic behavior and physical phenomena during the laser cleaning process. This study addresses the gap in laser cleaning of lubricating coatings on forgings and provides a theoretical foundation for the subsequent application of laser cleaning on the surface lubricating coatings of formed workpieces.

## 2. Materials and Methods

### 2.1. Materials and Samples Preparation

**Figure 1:**
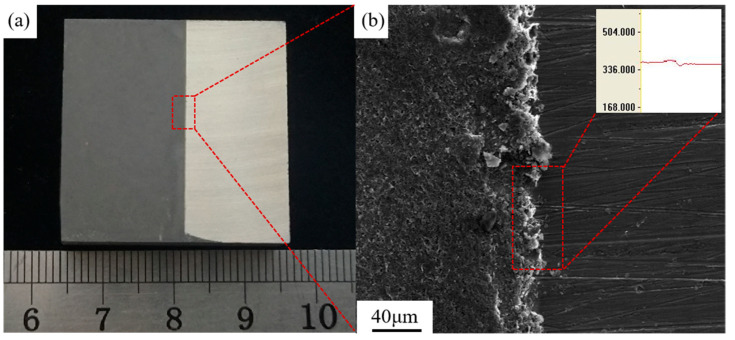
Surface morphology of the sample with the graphite lubricating coating at: (**a**) Macro level; (**b**) Microscopic level; The inset shows the measure result of the coating thickness using a confocal laser microscope.

In this experiment, MB15 magnesium alloy samples with dimensions of 20 mm × 20 mm × 4 mm (length × width × thickness) were prepared, and their main elemental composition is shown in Table 1. The samples were meticulously polished using sandpaper and thoroughly cleaned with alcohol to ensure surface cleanliness. The samples were heated in a furnace to around 110 °C, then water-based graphite emulsion was sprayed on them. After the water in the atomized graphite evaporated, the graphite adhered to the surface of the magnesium alloy, forming the graphite lubricating coating of 15 μm. The macro and micromorphology of the sample surface and coating thickness are shown in Figure 1.

### 2.2. Laser Cleaning Experimental Equipment

**Figure 2:**
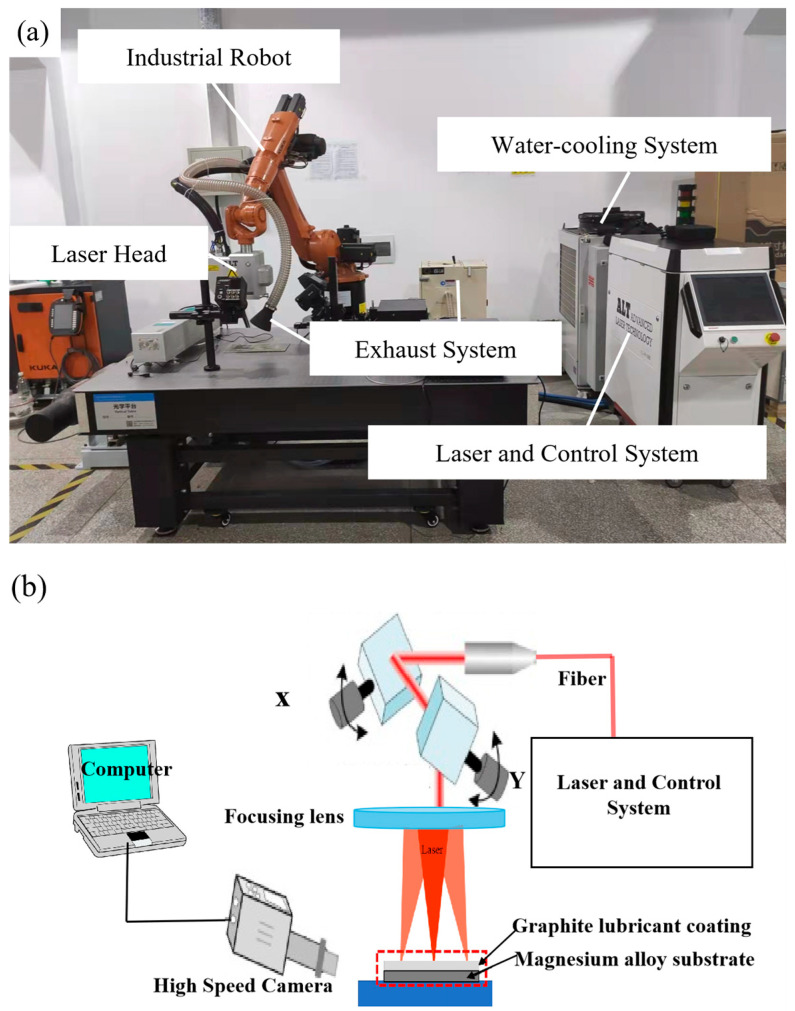
(**a**) Laser cleaning device; (**b**) Schematic diagram of laser cleaning device principle.

The pulsed laser cleaning device used in this experiment is shown in Figure 2a, which consists of five parts: nanosecond fiber laser, scanning galvanometer, control system, industrial robot, and dust removal system. Its schematic diagram is shown in Figure 2b. A nanosecond pulsed fiber laser (YLPN-100-30×100-1000, IPG photonics, Marlborough, MA, USA) with a maximum average power of 1000 W was employed in the experiments, which operated at a laser wavelength of 1064 nm, a spot diameter of 1 mm, and a maximum laser scanning range of 100 mm × 100 mm. The detailed laser cleaning parameters are listed in Table 2. Experimental samples were fixed on an X-Y horizontal platform, and the position of the light output can be adjusted to align with the sample using a robotic arm. Laser light was transmitted through optical fibers and directed by a two-dimensional galvanometer scanning head to conduct Z-shaped reciprocating scanning on the X-Y plane, aiming to clean the specified area of the target plane.

### 2.3. Characterization Methods

Metallographic microscopy (HAL-100, Carl Zeiss AG, Oberkochen, Germany) and scanning electron microscopy (Quanta 200FEG, FEI, Hillsborough, OR, USA and SU5000, HITACHI, Tokyo, Japan) were used to observe the microstructure of the cleaned sample surface. X-ray energy-dispersive spectroscopy (EDS, ULTIMATELY MAX40, Oxford Instruments, Oxford, UK) was used to analyze the elemental content of the cleaned sample surface. A laser scanning confocal microscope (OLS-3000, HITACHI OLYMPUS, Tokyo, Japan) was used to measure the surface roughness of the cleaned sample and observe the 3D surface morphology. High-speed cameras (FASTCAM Mini UX 100, PHOTRON, Tokyo, Japan) were utilized to monitor in real time the dynamic behavior of the decomposition and peeling of lubricating coatings on the substrate surface during laser cleaning.

## 3. Results

### 3.1. Laser Cleaning Process and Mechanism

When a pulsed laser irradiates the surface of the material to be cleaned, it interacts with the material. Part of the laser that strikes the sample is reflected and transmitted, while the rest is absorbed by the sample surface. At the microscopic level, the laser energy is absorbed by electrons, atoms, and lattice vibrations, leading to changes in energy. At the macroscopic level, this manifests as surface material gasification, combustion, melting, and explosive debris splashing, all of which are accompanied by the removal of surface contaminants. This study used a high-speed camera to capture real-time dynamic behavior during laser cleaning and recorded the dynamic process of pulsed laser acting on the surface of the sample.

**Figure 3:**
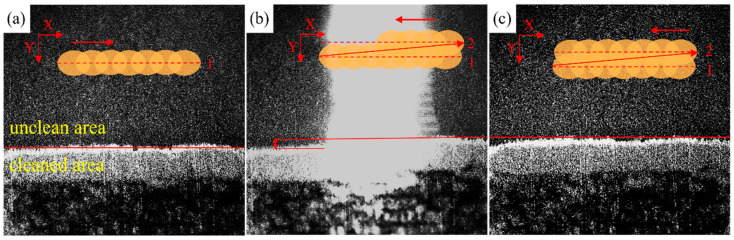
The process of laser cleaning: (**a**) laser spot completes scanning path 1; (**b**) laser spot is moving; (**c**) laser spot completes scanning path 2. The dashed lines indicate the laser scanning paths, and the arrows indicate the scanning directions.

During the laser cleaning process, the light spot is output to the surface to be cleaned. Figure 3 shows the process of the laser spot walking on the surface of the sample. In the image, the area above the red line annotation is the uncleaned area, while the area below the red line represents the cleaned area. Comparing between Figure 3a,c, it can be observed that the laser has a significant cleaning effect on the area it has traveled through. Compared to the already cleaned area in Figure 3a, the area in Figure 3c that has been completely cleaned has increased. Figure 3b shows the phenomenon of the light spot walking, and it can be clearly seen in the figure that the area scanned to the left by the light spot has a significant cleaning effect, exposing a bright magnesium alloy substrate. Based on the laser being used as a reference, comparing the cleaned area on the right and the uncleaned area on the left, it can be concluded that laser cleaning can effectively remove the graphite lubricating film coating on the surface of the magnesium alloy.

At present, the interaction mechanism between laser and materials can be divided into photothermal effect, photochemical effect, and photophysical effect. Usually, the removal of materials is caused by the combined action of several mechanisms. This study selected three representative cleaning processes—namely, partial removal (1.27 J/cm^2^), complete removal (3.82 J/cm^2^), and substrate damage (6.37 J/cm^2^)—for high-speed camera observation to analyze the dynamic behavior of coating peeling and reveal the mechanism of laser cleaning of graphite lubricating coating on the magnesium alloy surface. The laser is irradiated onto the surface to be cleaned, causing the coating to absorb energy, increase in temperature, and undergo thermal conduction. As the coating temperature rises to a certain value, a series of physical and chemical reactions such as melting, evaporation, and combustion will occur. Large blocks of material are transformed into small blocks or molecules, causing the coating to detach from the substrate surface. The increase in temperature is positively correlated with the laser fluence, and the larger the laser fluence the higher the instantaneous temperature of the coating, and the more severe the removal phenomenon.

**Figure 4:**
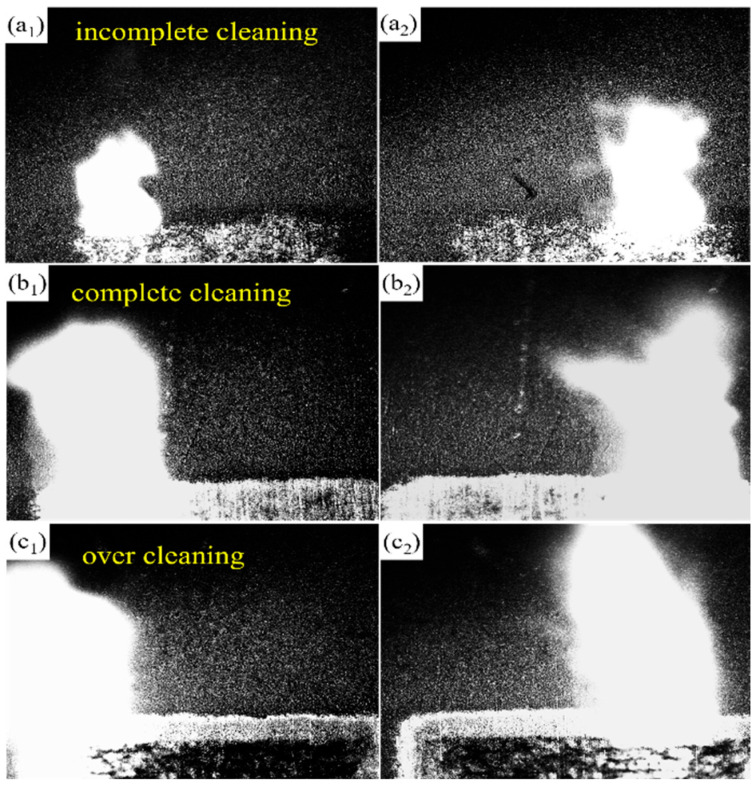
Laser cleaning dynamic behavior: (**a_1_**,**a_2_**) 1.27 J/cm^2^; (**b_1_**,**b_2_**) 3.82 J/cm^2^; (**c_1_**,**c_2_**) 6.37 J/cm^2^.

As shown in Figure 4, as the laser fluence increases, the laser cleaning effect improves and the reaction becomes more violent [33,34]. During the cleaning process captured by high-speed cameras, obvious thermal ablation behavior can be observed mainly manifested in two forms: selective evaporation and peeling of the evaporation reaction. After the laser spot moves, a large amount of smoke, dust, and debris can be observed. And as the laser fluence increases, the combustion becomes more intense and the amount of debris splashing around increases. The coating absorbs laser energy, causing various physical and chemical changes, including gasification, ablation, and peeling, to remove the graphite coating together [30,35,36]. When the laser fluence is 1.27 J/cm^2^, although the cleaning threshold of graphite coating is reached, the energy is not enough, and only a part of the graphite film coating is removed. As the laser energy increases, the graphite coating absorbs more energy, the physical and chemical reactions become more violent, and the graphite film coating is completely removed.

### 3.2. The Effect of the Laser Fluence on Cleaning

The above analysis of the laser cleaning process has concluded that laser cleaning technology can effectively remove graphite lubricating coatings on the surface of magnesium alloys. Laser cleaning technology has application and research value for removing the lubricating coating on the surface of hot formed workpieces.

To study the laser cleaning characteristics of graphite lubricating coatings and determine the optimal process parameters, the single-variable method was used to investigate the influence of three important parameters: laser fluence, pulse frequency, and scanning speed on the cleaning efficacy.

**Figure 5:**
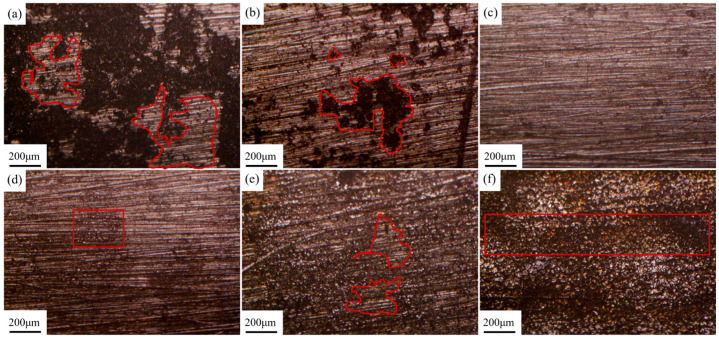
Macromorphology of sample surface after laser cleaning at different laser fluences: (**a**) 1.27 J/cm^2^; (**b**) 2.55 J/cm^2^; (**c**) 3.82 J/cm^2^; (**d**) 5.09 J/cm^2^; (**e**) 6.37 J/cm^2^; (**f**) 7.64 J/cm^2^.

**Figure 6:**
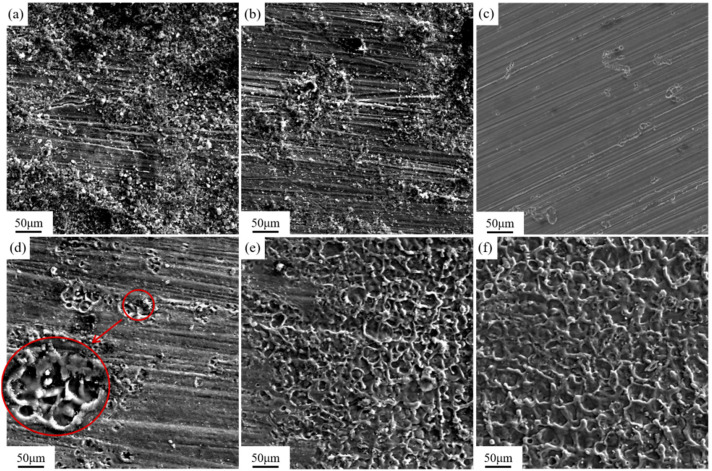
Microscopic morphology of sample surface after laser cleaning at different laser fluences: (**a**) 1.27 J/cm^2^; (**b**) 2.55 J/cm^2^; (**c**) 3.82 J/cm^2^; (**d**) 5.09 J/cm^2^; (**e**) 6.37 J/cm^2^; (**f**) 7.64 J/cm^2^.

Laser fluence (*F*) is an important parameter affecting cleaning quality, which can be coupled with laser power, spot diameter, and pulse frequency parameters. The laser fluence (*F*) density can be expressed by the Formula (1) [15]:

(1) $$F=\frac{{4P}_{v}}{\pi fd^{2}}$$

where *P_v_* is the average laser power, *f* is the pulse frequency, and *d* is the diameter of the focused spot. The pulse frequency used in this experiment is 10 kHz and the diameter of the focused spot is 1 mm. Figure 5 shows the surface morphology of the sample after laser cleaning at different laser fluences. As shown in Figure 5a, there is a large amount of graphite residue on the surface of the sample with a laser fluence of 1.27 J/cm^2^. The majority of the sample surface is still obscured by the black graphite lubrication coating, with only certain areas (indicated by the circled regions in the figure) revealing the magnesium alloy morphology. As the laser fluence escalates from 2.55 to 5.09 J/cm^2^, the cleaning efficacy of the coating markedly improves. As shown in Figure 5b–d, the residual amount of graphite was reduced from the local blocky residue in Figure 5b to no obvious residue observable by the naked eye in Figure 5c and Figure 6d, and the magnesium alloy substrate was exposed on the surface of the samples after cleaning. However, partial melting traces are observed in some scratch areas in Figure 5d. Figure 5e,f depict the cleaning outcomes at laser fluences of 6.37 J/cm^2^ and 7.64 J/cm^2^, respectively. At these higher fluences levels, while the graphite is entirely eliminated, excessive laser energy adversely impacts the substrate. In Figure 5e, melt marks are evident across much of the sample surface, with localized areas exhibiting a yellowish burn. Meanwhile, in Figure 5f, the entire matrix is covered by melt marks, accompanied by regular parallel stripe-like laser overlap marks This phenomenon indicates that the surplus laser energy has caused synchronous surface treatment on the exposed magnesium alloy substrate surface after removing the graphite coating within the laser range.

Figure 6 shows the microstructure of the magnesium alloy surface after cleaning with different laser fluences observed under a scanning electron microscope. Compared with Figure 6a–f, as the laser fluence increases, the cleaning efficacy of the coating gradually improves. As shown in Figure 6c, the graphite is completely removed at 3.82 J/cm^2^, revealing the morphology of the substrate, and the substrate is not affected by the laser. At 5.09 J/cm^2^, although the graphite was completely removed, the magnesium alloy substrate began to appear slight melting marks at the scratch, indicating that the excess laser energy was also absorbed by the substrate surface, while the laser removed the graphite coating. When the laser is radiated to the surface of the sample, the flatness of the sample’s surface will exert an impact on the absorption of light. The reflectivity at smooth positions is relatively higher. Because at the areas where scratches concentrate, the laser will undergo multiple reflections and absorptions within the grooves of the scratches. The absorption efficacy of the laser at these locations is relatively higher. The additional absorbed energy causes some parts of the scratched areas to reach the melting point first, resulting in the appearance of melting marks. Compared to other areas, it first reached the melting point of the magnesium alloy and appeared melting phenomenon. Figure 6e,f shows that when the laser energy is sufficiently high, the substrate surface absorbs enough energy, resulting in large-scale melting. The substrate surface reaches the melting temperature and then rapidly cools, resulting in an obvious cloud-like cooling and melting morphology on the metal surface. This indicates that the substrate has been damaged by excess laser energy, and the laser fluence at this time is too high. The process of laser cleaning shown in Figure 3 also proves this point. As shown in Figure 3, when the laser energy is too high, the area of excessive cleaning also increases with the increase of the laser scanning path. Due to the sufficient laser energy, the surface coating has been removed after scanning path 1. After path 2 moves, the originally cleaned area is damaged due to the overlapping of two adjacent light spots in the Y direction, and the exposed substrate absorbs excess energy, resulting in obvious black marks in high-speed photography images.

**Figure 7:**
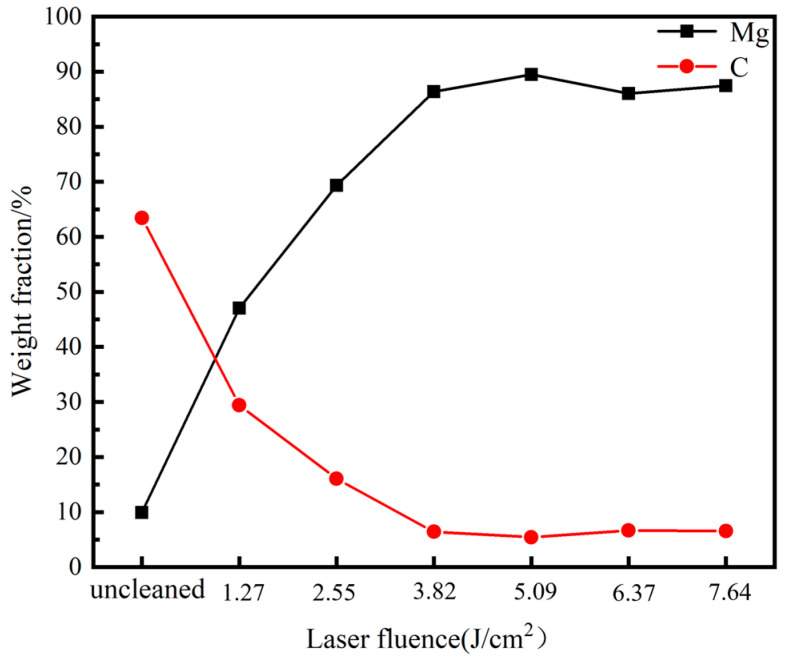
Surface element content of cleaned samples under different laser fluences.

Figure 7 shows the EDS scanning results of the cleaned sample surface at different average powers. The surface of the uncleaned sample is composed of C (~63.43 wt%)—the main component of the graphite coating, O (~25.42 wt%) and Mg (~9.0 wt%), Zn, Si, and other elements. This study defines clearance rate as the percentage of the difference in surface graphite content before and after cleaning to the initial surface graphite content. As the average power increases from 1.27 to 1.27 J/cm^2^, the C content decreases, while the Mg content gradually increases. Within the range of 1.27~3.82 J/cm^2^, the C content exhibits a significant downward trend, decreasing from 63.43% before cleaning to 6.42%, the clearance rate reaches 89.88% at 3.82 J/cm^2^. These data indicate that increasing laser fluence within this range significantly improves the cleaning efficacy of the lubrication coating. The curve tends to stabilize within the range of 3.82 to 7.64 J/cm^2^, and the clearance rate remains at around 90%. The cleaning efficacy is optimal at the laser fluence of 5.09 J/cm^2^, achieving a clearance rate of 91.46%, slightly higher than 89.88% at 3.82 J/cm^2^.

**Figure 8:**
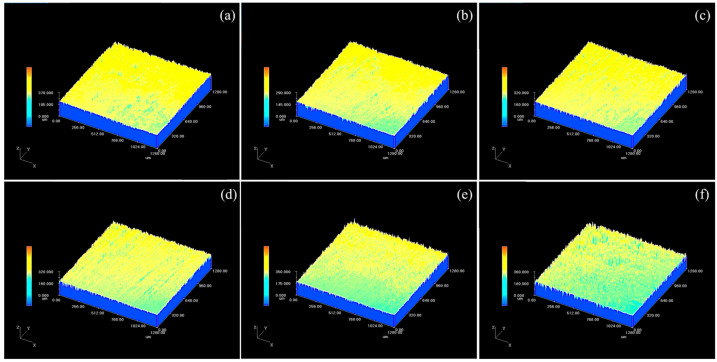
3D Micromorphology of the sample surface after cleaning with different laser fluences: (**a**) 1.27 J/cm^2^; (**b**) 2.55 J/cm^2^; (**c**) 3.82 J/cm^2^; (**d**) 5.09 J/cm^2^; (**e**) 6.37 J/cm^2^; (**f**) 7.64 J/cm^2^.

**Figure 9:**
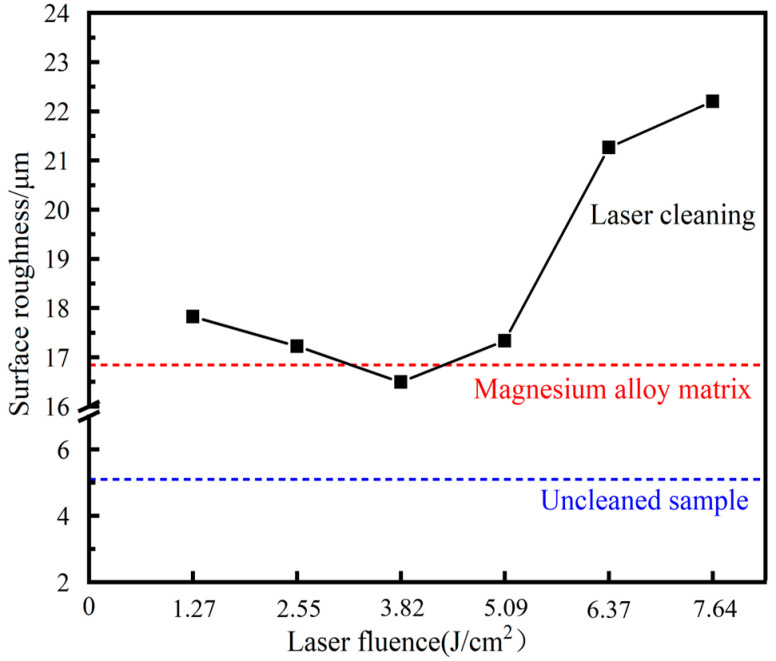
Surface roughness of samples under different laser fluences.

Laser action can alter the microstructure of the substrate surface, which can impact the properties of the magnesium alloy substrate. Figure 8 displays the three-dimensional microstructure of the sample surface after cleaning with various laser fluences. Figure 9 illustrates the impact of laser fluence on the surface roughness of the cleaned sample. Surface roughness refers to the unevenness of a machined surface with relatively small intervals and tiny peaks and valleys. In this paper, the description of the surface roughness value refers to the arithmetic mean deviation Ra of the contour, and its measurement principle is the arithmetic mean of the absolute value of the contour deviation distance within the sampling length. As the laser fluence increases from 1.27 to 3.82 J/cm^2^, the surface roughness of the sample decreases. However, as the laser fluence further increases from 3.82 to 7.64 J/cm^2^, the surface roughness of the sample increases. The surface roughness of the 3.82 J/cm^2^ cleaned sample is almost the same as that of the original magnesium alloy substrate surface, indicating that the cleaning efficacy is optimal at 3.82 J/cm^2^. The graphite has been completely removed without causing any damage to the substrate. The surface roughness of the original substrate and the cleaned sample is significantly higher than that of the uncleaned sample. This phenomenon is due to a large number of scratches on the substrate caused by artificial sandpaper polishing, and the sprayed graphite particles adhere to the surface of the substrate to fill in the uneven scratches, making the surface flat. When the laser fluence is low, the laser cleaning efficacy is poor, and the remaining large graphite on the sample’s surface increases the wave peak, resulting in higher surface roughness. As the laser fluence increases, the amount of residual graphite on the sample surface gradually decreases, leading to a reduction in roughness value. When the laser energy is too high, the excess energy is absorbed by the substrate, causing surface melting. Moreover, the higher the laser fluence, the more severe the melting becomes. Due to the short duration of pulsed laser action, the molten metal cools down before it can flow rapidly, leading to increased laser fluence and higher roughness.

Based on the above analysis, as the laser fluence increases, the cleaning effect of the film coating gradually improves. However, it is not true that higher laser fluence always leads to better results. When the damage threshold of the substrate is exceeded, high-energy laser will cause damage to the substrate. Combining the cleaning effect and non-destructive considerations, the most suitable laser fluence for a 15 μm thick graphite lubricating coating on the surface of magnesium alloys is 3.82 J/cm^2^.

### 3.3. The Effect of the Pulse Frequency on Cleaning

Pulse repetition frequency refers to the number of regular laser pulses output per unit time, which is the number of times pulses repeat within 1 second. When the laser power *P* is constant, the single-pulse energy *P_S_* can be calculated based on the pulse frequency *f*, as shown in the Formula (2):

(2) $$P_{S}=\frac{P}{f}$$

At a fixed laser power (*P*), the higher the pulse frequency (*f*), the smaller the output single-pulse energy (*P_S_*).

**Figure 10:**
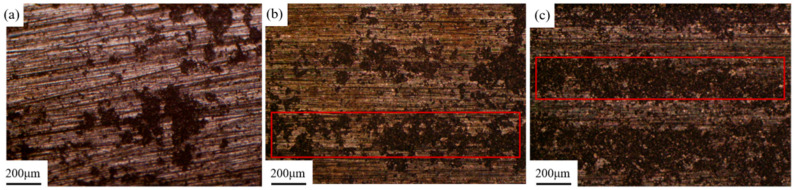
Macromorphology of the sample surface after laser cleaning at different pulse frequencies: (**a**) 10 kHz; (**b**) 20 kHz; (**c**) 30 kHz. The area circled in red is strip shaped residual graphite traces after laser spot scanning.

Figure 10 shows the surface morphology of the samples after laser cleaning at different pulse frequencies. Three pulse frequencies of 10 kHz, 20 kHz, and 30 kHz were selected for laser cleaning experiments. In Figure 10a, it can be observed that after cleaning at 10 kHz, most of the bright magnesium alloy matrix is exposed on the surface of the sample, indicating a more effective cleaning outcome. In Figure 10b, after cleaning at 20 kHz, there is a significant increase in residual graphite, and the cleaned area shows no apparent metallic luster. As shown in Figure 10c, after cleaning at 30 kHz, the surface displays distinct rows of laser spot motion traces. The residual strip of graphite at the overlap of the two rows has not been removed, and the residual graphite coating in the area where the center energy of the laser spot moves higher is thicker than at 20 kHz.

**Figure 11:**
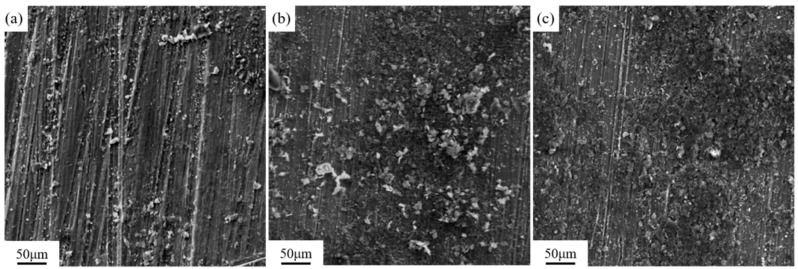
Microscopic morphology of sample surface after laser cleaning at different pulse frequencies: (**a**) 10 kHz; (**b**) 20 kHz; (**c**) 30 kHz.

Figure 11 shows the microstructure of the magnesium alloy surface after cleaning with different pulse frequencies observed under a scanning electron microscope. The information shown in Figure 11 is the same as that in Figure 10. As the pulse frequency increases, the cleaning efficacy of the membrane coating gradually decreases.

**Figure 12:**
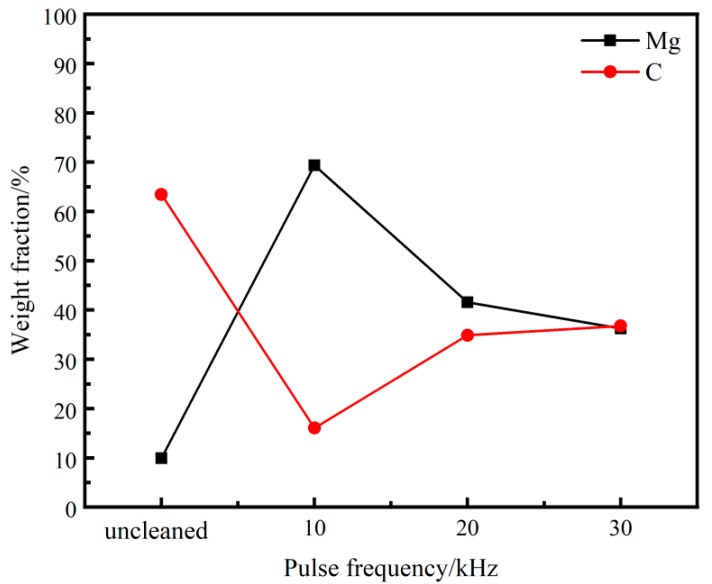
Surface element content of cleaned samples under different pulse frequencies.

Figure 12 shows the EDS scanning results of the cleaned sample surface at various pulse frequencies. As the pulse frequency increases from 10 to 30 kHz, the C content increases, while the Mg content decreases gradually. Under the pulse frequency of 10 kHz, the C content on the surface of the sample is 16.05%, and the clearance rate is 74.70%, which is 32.63% higher than that at 30 kHz. According to Figure 12, reducing the pulse frequency can significantly enhance the cleaning efficacy of the lubrication coating.

**Figure 13:**
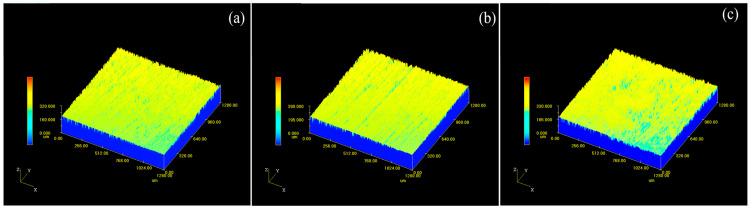
3D Micromorphology of sample surface after cleaning with different pulse frequencies: (**a**) 10 kHz; (**b**) 20 kHz; (**c**) 30 kHz.

**Figure 14:**
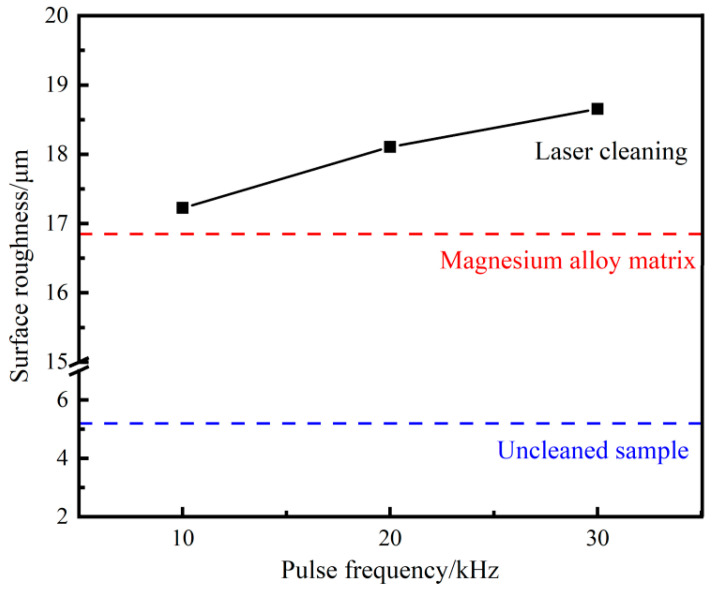
Surface roughness of samples under different pulse frequencies.

Figure 13 shows the three-dimensional microstructure of the sample surface after cleaning with different pulse frequencies, while Figure 14 shows the effect of pulse frequency on the surface roughness of the cleaned sample. As the pulse frequency increases in the range of 10–30 kHz, the surface roughness of the sample increases and is higher than the original matrix, significantly higher than the surface of the uncleaned sample. When the laser power is constant, as the pulse frequency increases, the energy of a single laser pulse decreases, which is not enough to remove all lubricating coatings. The residual graphite on the surface of the sample leads to a decrease in surface smoothness and an increase in roughness.

Based on the above analysis, as the pulse frequency increases, the cleaning efficacy of the membrane coating gradually decreases. Combining the cleaning effect and non-destructive considerations, the most suitable pulse frequencies for a 15 μm thick graphite lubricating coating on the surface of magnesium alloys is 10 kHz.

### 3.4. The Effect of the Scanning Speed on Cleaning

Scanning speed refers to the speed at which the laser moves laterally along the X direction. The faster the scanning speed, the less time is required for scanning in the X direction, and the lower the overlap rate of the light spot in the X direction. The slower the scanning speed, the more time is required for scanning in the X direction, and the higher the overlap rate of the light spot in the X direction.

**Figure 15:**
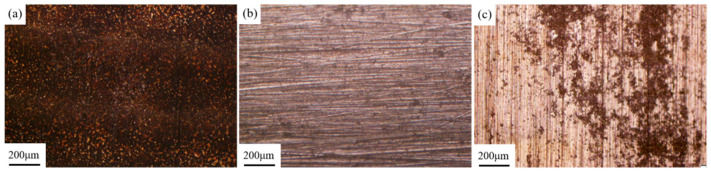
Macromorphology of sample surface after laser cleaning at different scanning speeds: (**a**) 1000 mm/s; (**b**) 3000 mm/s; (**c**) 5000 mm/s.

Figure 15 displays the macroscopic morphology of the sample surface after laser cleaning at various scanning speeds. Three scanning speeds of 1000 mm/s, 3000 mm/s, and 5000 mm/s were selected for cleaning experiments with a fixed laser fluence of 3.82 J/cm^2^ and a pulse frequency of 10 kHz. Comparing Figure 15a–c, it can be seen that the cleaning efficacy is best at 3000 mm/s. The graphite coating on the surface of the sample can be completely removed, revealing the original substrate’s bright morphology. At a speed of 1000 mm/s, clearly visible rows of laser motion marks appear on the surface, and the intersection line is burnt black. When the scanning speed is 5000 mm/s, a significant amount of residual graphite can be observed on the cleaned surface.

**Figure 16:**
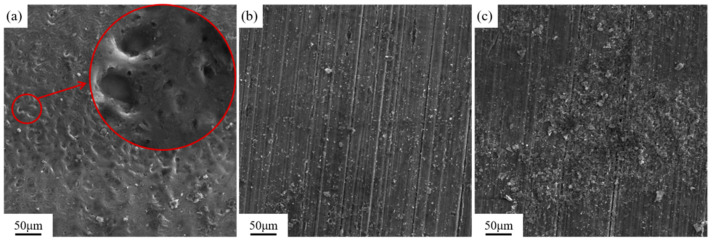
Microscopic morphology of sample surface after laser cleaning at different scanning speeds: (**a**) 1000 mm/s; (**b**) 3000 mm/s; (**c**) 5000 mm/s.

Figure 16 shows the microstructure of the sample surface after cleaning at different scanning speeds. In Figure 16a, a too-low speed (1000 mm/s) resulted in a high overlap rate of X-direction light spots, and the substrate surface absorbed too much residual energy, resulting in a large area of melting phenomenon. Obvious striped laser marks were seen in the microscopic morphology image. The best cleaning efficacy was achieved at a scanning speed of 3000 mm/s, where the graphite was completely removed and the substrate was not affected by the laser. At 5000 mm/s, the overlap rate of the X-direction spot was too low, and the laser energy acting on the unit area was small, with obvious traces of graphite residue visible on the surface. Based on the above analysis, it can be concluded that as the scanning speed decreases, the cleaning efficacy of the film coating gradually improves. However, too low a speed will lead to a high overlap rate of the laser spot in the X-direction, and the laser energy in the overlap area will be too high, damaging the substrate.

**Figure 17:**
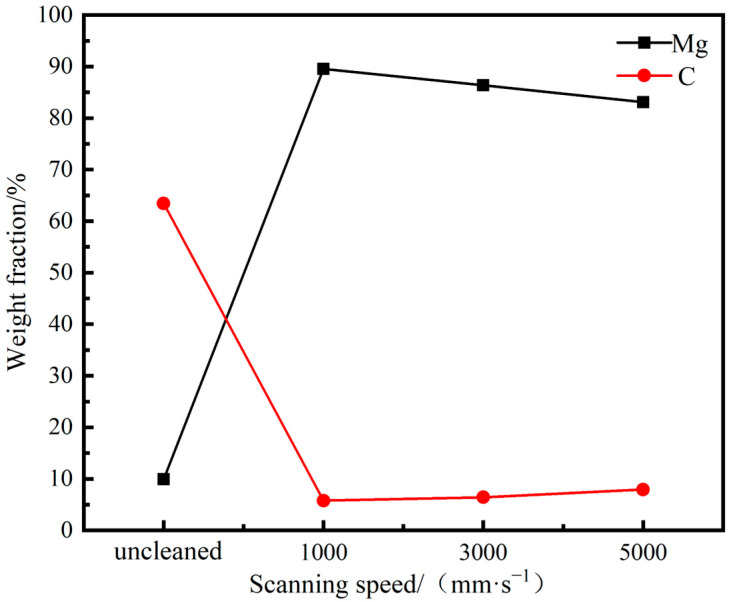
Surface element content of cleaned samples under different scanning speeds.

Figure 17 shows the EDS scanning results of the cleaned sample surface at different scanning speeds. The results indicate that as the scanning speed increases from 1000 to 5000 mm/s, the C content increases, while the Mg content decreases gradually. Under the scanning speed of 1000 mm/s, the content of C element decreased from 63.43% before cleaning to 5.77%, and the clearance rate reached 90.90%. The clearance rate was 89.88% at 3000 mm/s and 89.83% at 5000 mm/s. It can be seen that under the condition of an average power of 300 W, the scanning speed has little effect on the cleaning efficacy. As the scanning speed increases from 1000 to 5000 mm/s, the clearance rate decreases, but the slope is gentle and remains around 90%. Compared to the significant impact of average power and pulse frequency on the surface element content of the sample, the scanning speed is not considered to affect the cleaning efficacy of the lubricating coating significantly. Therefore, it is not the main focus of discussion.

Therefore, based on all the above analyses and considering the influence of laser parameters on surface morphology, element content, and roughness, as well as the cleaning effect and whether the substrate is damaged, it can be concluded that *F* = 3.82 J/cm^2^, *P* = 300 W, *f* = 10 kHz, and *v* = 3000 mm/s are most suitable for cleaning the 15 μm thick graphite lubricating coating on the surface of the MB15 magnesium alloy.

## 4. Conclusions

This study conducted laser cleaning experiments on the graphite lubrication coating on the surface of magnesium alloy using a nanosecond pulsed laser. The influence of laser power, pulse frequency, and scanning speed on the surface morphology, element content, and surface roughness of cleaned samples were studied. The innovation of this study was to verify the feasibility of using a nanosecond pulsed laser to clean the lubricating coating on the surface of forged specimens and elucidate the main mechanism of laser cleaning the coating. The main results are as follows:

(1) As the laser fluence increases, the energy density gradually increases, and the clearance rate improves. As the pulse frequency increases, the single pulse energy decreases and the clearance rate decreases. The scanning speed increases from 1000 to 5000 mm/s, and the spot overlap rate gradually decreases, while the clearance rate first increases and then decreases.

(2) Increasing laser power and reducing pulse frequency can significantly reduce C content and improve cleaning efficacy. The scanning speed (1000~5000 mm/s) has little effect on the changes in element content, and the C content has remained around 6%.

(3) Under the action of *F* = 3.82 J/cm^2^, *f* = 10 kHz, and *v* = 3000 mm/s, the graphite clearance rate reached 89.88% and the matrix was not damaged, which is beneficial for cleaning the 15 μm thick graphite lubricating coating of the MB15 magnesium alloy surface.

(4) The dominant mechanism for laser cleaning of graphite lubricating coatings on magnesium alloy surfaces is thermal ablation mechanism, and the higher the laser fluence, the more intense the ablation phenomenon and the better the cleaning effect.

## Figures and Tables

**Table 1:** MB15 magnesium alloy chemical composition (wt%).

Al	Mn	Zn	Zr	Cu	Ni	Si	Fe	Be	Mg
≤0.050	≤0.100	5.000~6.000	0.300~0.900	≤0.050	≤0.005	≤0.050	≤0.050	≤0.010	Bal

**Table 2:** Laser cleaning parameters.

Parameter	Symbol	Value	Unit
Wavelength	*λ*	1064	nm
Average laser power	*p*	<1000	W
Spot diameter	*D*	1	mm
Pulsed width	*τ*	30, 40, 60, 100	ns
Pulse frequency	*f*	≤50	kHz
scanning speed	*v*	≤5000	mm/s
Overlap rate	*O*	50–90	%

## Data Availability

The original contributions presented in this study are included in the article. Further inquiries can be directed to the corresponding author.
